# Japan’s Ethical Guidelines for Epidemiologic Research: A history of their development

**DOI:** 10.2188/jea.15.107

**Published:** 2005-08-30

**Authors:** Takeo Nakayama, Michi Sakai, Brian Taylor Slingsby

**Affiliations:** 1 Department of Health Informatics, Kyoto University School of Public Health.; 2 Department of Medical Epidemiology, Kyoto University School of Public Health.; 3 Department of Biomedical Ethics, Graduate School of Medicine, University of Tokyo.

**Keywords:** Epidemiology, Ethics, Guidelines, Information Management, Informed Consent

## Abstract

During the latter half of the 1990s, Japanese healthcare professionals and policy-makers recognized the value of an “evidence-based” approach. At the same time, an increased social awareness of the need to protect research participants and personal information began to appear. Recognition of an evidence-based approach further promoted epidemiologic research while regulations on personal information protection imposed certain limitations on this same research. In April 2000, as a solution to this conflict, a working group funded by Japan’s Ministry of Health and Welfare (MHW; currently the Ministry of Health, Labour and Welfare: MHLW) proposed a first draft of ethical guidelines for epidemiologic research. Over the next two years, the collection of domestic and foreign data by working groups and governmental ad hoc committees, questions raised by the mass media, and public statements made by organizations, such as the Science Council of Japan and the Japan Epidemiologic Association (JEA), led to a collaborative effort between the Ministry of Education, Culture, Sports, Science and Technology and the MHLW. This effort led to the creation of the Ethical Guidelines for Epidemiologic Research in June 2002, which was revised in 2004. Furthermore, JEA also announced the Ethical Guidelines for Conducting of Epidemiologic Research in October 2002. While the development of these ethical guidelines has been a challenge for Japanese epidemiologists, it has also allowed the epidemiologic community to understand their role in society. This review aims to provide insight into the interaction between the epidemiologic community and society by assessing historically the developmental process of these ethical guidelines.

In June 2002, Japan’s Ethical Guidelines for Epidemiologic Research were announced as the culmination of a four-year project by epidemiologists, clinicians, ethicists, experts in law, and the media as representatives of the general public.^1^ In this report, we review the events that contributed to these published guidelines and, in particularly, how governmental ministries and the Japan Epidemiological Association (JEA) played a role in the development of these published guidelines.

Currently in Japan, there exist two sets of ethical guidelines for epidemiologic research: a national governmental set of guidelines and the JEA set of guidelines. While the JEA guidelines, which were announced in October 2002, apply to all JEA members, the governmental guidelines apply to all studies that employ epidemiologic methodology.

Even though the governmental guidelines were revised in December 2004, this review primarily focuses on the first governmental ones issued in 2002 and describes a history of events that led up to them.

## PART I: An initial JEA survey and its history (1991-1996)

The year of 1991 was a milestone year for epidemiology, particularly in Japan. In January, the JEA held its first scientific meeting at the National Cancer Center, Tokyo. Second, the Council for International Organizations of the Medical Sciences (CIOMS) released its international guidelines for the ethical review of epidemiologic studies.^2^ They were immediately translated into Japanese by a lawyer, Tadahiro Mitsuishi.^3^ Since then, several Japanese epidemiologists have examined the ethical implications of epidemiologic research.^4^[Table tbl01]

**Table 1. tbl01:** A brief history of events related to Japan’s ethical guidelines for epidemiologic research (1980-2003).

Calendar year	Month	Event
1980	September	In Europe, the OECD proposing recommendation of the council concerning guidelines governing the protection of privacy and transborder flows of personal data
1982		The CIOMS releasing the international ethical guidelines for biomedical research involving human subjects
1989		Establish of a three year project on the ethical issues in preventive medicine (Principle Investigator: Shunichi Yamamoto)
1990		Formation of the research project funded by the Ministry of Labour on the prevention of work-related diseases including cardiovascular diseases in Japan
1991	January	**The JEA holding its first scientific meeting at the National Cancer Center in Tokyo** **The CIOMS releasing the international guidelines for ethical review of epidemiologic studies** **In Canada, Guyatt publishing a report entitled “Evidence-based Medicine” in the ACP (American College of Physician) Journal Club**
1993		A survey conducted with the purpose the process of informed consent in the large-scale study “The Collaborative Cohort Study for Evaluation of Cancer Risk” (The survey results were later reported at the IEA conference in Nagoya in August 1996) The CIOMS revising the international ethical guidelines for biomedical research involving human subjects
1995	November	**The European Union Directive 95/46/EC established on the protection and free movement of personal data**
1996	January	**The JYES held the first conference on “Informed consent in epidemiologic research” at the JEA 6th Scientific Meeting in Nagoya**
	August	**The IEA Conference held in Nagoya**
1997	January	The 2nd JYES conference held in Tokyo on “Informed consent in epidemiologic research,” at the 7th scientific meeting of the JEA
1998	January	**The 3rd JYES conference held in Tokyo on “Informed Consent in Epidemiologic Research,” at a joint coherence (2nd Asia-Pacific Congress of Epidemiology and 8th Scientific JEA Meeting)**
	April	**Formation of the MHLW Health Sciences Working Group on “Development of Ethics Guidelines for Informed Consent in Epidemiologic Research” (Principal Investigator: Akiko Tamakoshi, Tamakoshi Group)**
1999	May	**The Health Science Council presented “The MHW future modality towards the 21st Century”**
	July	The Advanced Information and Telecommunications Society Promotion Headquarters (in Japanese, *Kodo joho tsushin shakai suishin honbu*), led by the prime minister, established an investigating committee on personal information protection
	November	Advanced Information and Telecommunications Society Promotion Headquarters Meeting on Personal Information Protection, “The modality of a personal information protection system in Japan (in Japanese, *Wagakuni niokeru Kojin joho hogo sisutemu no* *arikata nitsuite*)” (Interim Report) The Asahi Shimbun publishing, “*Jumin no idenshi mudctnkaiseki: koketsuatsu no kenkyuyo ni kenshin no saiketsu tsukau*” (Genetic analysis of citizen DNA without permission: Use of blood taken at health checkups for a research on high blood pressure)
	December	Prime Minister Keizo Obuchi presenting the Millennium Project which includes “the promotion of information infrastructure in education,” “the realization of e-government” and “research on the human genome”
2000	January	JEA setting up the ad hoc committee on ethical issues (Chair: Yutaka Inaba). Formation of the MHLW Health Sciences Working Group on “Assessment of epidemiologic research in terms of contributions to health policy” (Principle investigator: Takeo Nakayama, in Japanese, *Ekigaku kenkyu no gyoseiteki sokumen karano hyouka ni kansuru kenkyu*)
	February	**Establishment of the National Committee in charge of the Personal Information Protection Bill. “Hearing” from parties of interest (March through August 2000)**
	March	**National Cancer Center Symposium, public symposium (e.g. personal information protection in epidemiologic research such as cancer registries)** The Advanced Medical Technology Assessment Panel of the MHLW Health Sciences Council and the *ad hoc* committee on privacy protection in research using epidemiologic methods beginning to draft a set of guidelines **The JEA releasing a public statement on the need to explain the relationship between epidemiologic research and personal information to the public**
	April	**A draft of Guidelines for Informed Consent in Epidemiologic Research (in Japanese, *Ekigaku kenkyu ni okeru infohmudo konsenio ni kansutu gaidorain*) Version 1.0 proposed by Tamakoshi Group** The JEA releasing a public statement on the legislature pertaining to personal information protection (Subcommittee <currently, Committee> on Ethical Issues : Chairperson, Yutaka Inaba) **Formation of the MHLW Health Sciences Working Group on study of ethical ramifications and personal information protection in research and programs using epidemiologic methods (Principle investigator: Eiji Maruyama, Maruyama Group)**
	October	**The National Committee in charge of the Personal Information Protection Bill announcing the basic principles of the future bill**
2001	March	The Science Council of Japan the 7th Division announcing “The modality of legislation on the protection of personal information from the perspective of medical research (in Japanese, *Igaku kenkyu kara mita kojin joho no hogo ni kansuru hosei no arikata ni* *tsuite*)” **AJMC creating a sub-committee to develop a set of ethical guidelines for epidemiologic research**
	April	The Maruyama Group presenting a draft of the Guideline for Bioethics and Privacy Protection in Epidemiologic Research (in Japanese, *Ekigaku teki shuho o mochiita kenkyu nado niokeru seimeirinri mondai oyobi kojin joho hogo no arikata ni kansuru shishin*) **The MHLW, MEXT, and METI publishing the Ethical Guideline for Human Genome and Gene Analysis Research** **The Diet stalling on enacting a law for personal information protection**
	May	The MHLW creating an *ad hoc* committee on the implementation of research using epidemiologic methods. This committee is under the jurisdiction of the Science and Technology Panel of the MHLW Health Sciences Council
	August	**The MEXT creating a sub-committee on research using epidemiologic methods and drafts ethics guidelines for epidemiologic research. This sub-committee belongs to the Bioethics and Safety Panel under the jurisdiction of the Council for Science** **and Technology** **Establishment of the IMJC for drafting a set of ethics guidelines**
	September	**The MEXT publishing the Guidelines for Derivation and Utilization of Human Embryonic Stem Cell**
2002	January	**The JEA announcing an ethical proclamation for conducting of epidemiologic research**
	June	The IMJC publishing the Ethical Guidelines for Epidemiologic Research (in Japanese, *Ekigaku kenkyu ni kansuru rinri shishin*)
	October	The JEA publishing the ethical guidelines for the conducting of epidemiologic research (in Japanese, *Ekigaku kenkyu o jisshi suruniatatteno rinri shishin*)” **The CIOMS revising the international ethical guidelines for biomedical research involving human subjects**
2003	May	**Enactment of the Personal Information Protection Act**
	July	The MHLW publishing the Ethical Guidelines for Clinical Studies
2004	December	**Revision of the Ethical Guidelines for Epidemiologic Research by the MHLW and MEXT (Enforced in April 2005)**
2005	January	**Revision of the Ethical Guidelines for Clinical Studies by the MHLW (Enforced in April 2005)**
	April	**Enforcement of the Personal Information Protection Act**

AJMC: Association of Japanese Medical Colleges

CIOMS: the Council for International Organizations of the Medical Sciences

IEA: International Epidemiological Association

IMJC: Inter-Ministry Joint Committee

JEA: Japan Epidemiological Association

JYES: Japanese Young Epidemiologists Society

METI: Ministry of Economy, Trade and Industry

MEXT: Ministry of Education, Culture, Sports, Science and Technology

MHLW: Ministry of Health, Labour and Welfare

OECD: Organization for Economic Cooperation and Development

In 2000, the Ministry of Health and Welfare (MHW) and the Ministry of Labour combined to form the Ministry of Health, Labour and Welfare (MHLW). The Ministry of Education, Science and Culture and the Science and Technology Agency combined to form the Ministry of Education, Culture, Sports, Science and Technology.

At the same time, Gordon Guyatt of McMaster University, Canada, published a noteworthy article, entitled “Evidence-based medicine,” in which he proposed several key concepts based on clinical epidemiology.^5^ This article initiated and promoted an EBM (evidence-based medicine) movement worldwide. Evidence of this rising boom was early seen in a research project conducted in Japan between 1990 and 1991 funded by the Ministry of Labour on the prevention of work-related diseases including cardiovascular diseases. This study was unique in that it was the collaborative project between physicians and epidemiologists. This project led several health policy makers and clinical researchers to recognize the significance of epidemiology. Under these circumstances, the formal introduction of EBM by governmental agencies in 1996^6^ stimulated a successive “epidemiology boom” among clinicians and policy-makers alike.

In Japan, several large community-based cohort studies were conducted throughout the 1980s and 1990s by mainly epidemiologists. Among them, one national collaborative cohort study, entitled “The Japan Collaborative Cohort Study (JACC Study) for Evaluation of Cancer Risk” was first funded in 1988 by the Ministry of Education, Science and Culture and the Science (currently the Ministry of Education, Culture, Sports, Science and Technology: MEXT).^7^ Several JEA researchers carried out a survey in 1993 on whether or not informed consent had been conducted in this study.^8^^,^^9^ The results of this survey, reported in 1996 at the International Epidemiologic Association (IEA), demonstrated that (1) informed consent for health questionnaires was carried out in all cohorts, but methods varied; (2) informed consent for the collection of blood was obtained in 28 of 34 cohorts (78%); and (3) this collaborative study group decided that specimens collected without consent should not be used. Combined, these results reflect a trend among many epidemiologists at that time: the primary concern was informed consent over protection of personal information.

## PART II: Discussion of the protection of personal information (1997-2000)

Following the JACC study, the Japanese Young Epidemiologists Society (JYES),^10^ a division of the JEA, continued to discuss the issue of informed consent in epidemiologic research at JEA conferences in 1996, 1997, and 1998. Their discussions resulted in the establishment of a working group on “Development of Ethics Guidelines for Informed Consent in Epidemiologic Research” (hereinafter refer to as the “Tamakoshi Group” after the principal investigator, Akiko Tamakoshi) in April 1998. The Tamakoshi Group, funded by the MHW, consisted primarily of epidemiologic researchers affiliated with the JYES and also included experts from the fields of bioethics and law.

Several significant events took place in Japanese society when the Tamakoshi Group was conducting their investigation (April 1998 - March 2000). Particularly beneficial to epidemiology was the MHW Health Sciences Council’s ranking of “the promotion of EBM and the utilization of information technology” as an area of focus in May 1999. Their acknowledgement emphasized the value of epidemiologic and clinical epidemiologic research, the basis of EBM. At the same time, the field of epidemiology was slightly strained by events leading up to the Personal Information Protection Bill. This bill was based on the a document published by the Organization for Economic Cooperation and Development (OECD) in 1980: The Recommendation of the Council Concerning Guidelines Governing the Protection of Privacy and Trans-Border Flows of Personal Data (hereinafter, the “Eight OECD Principles”).

In July 1999, the Advanced Information and Telecommunications Society Promotion Headquarters (in Japanese, *Kodo joho tsushin shakai suishin honbu*), led by the prime minister, established an investigating committee on personal information protection. In November 1999, their interim report, “Modality of the Personal Information Protection System in Japan (in Japanese, *Wagakuni niokeru kojin joho hogo shisutemu no arikata nitsuite*),” indicated a need to draft a basic law on personal information protection. The government formulated the national committee in February 2000 to submit the bill to the ordinary session of the Diet in spring 2001. This committee interviewed parties that were interested in this issue from February through August 2000 in a fashion of “hearing”.

At the same time, the field of epidemiology was hit hard by several newspaper articles on ethical problems related to epidemiologic research. A major Japanese newspaper published a story in November 1999 entitled “Genetic Analysis without Permission,” linking genetic analysis to the drawing of blood at mass screenings.^11^ Similar reports ensued, raising the level of social criticism regarding improper handling of personal information in epidemiologic research.^12^^,^^13^

Under these circumstances, the MHW organized several working groups such as a committee to assess the practical use of statistical data in the field of public health (chairman: Masahiro Takaishi, in Japanese, *Tokei joho no kodo riyo no seidoteki na arikata ni kansuru kentokai*). These working groups collected data primarily on foreign cancer registries while also assessing domestic problems. However, the contributions of epidemiologic research to society continued to go unrecognized by the public. In light of this, the MHW also funded a working group on the “Assessment of Epidemiologic Research in terms of Contributions to Health Policy” (principal investigator: Takeo Nakayama, in Japanese, *Ekigaku kenkyu no gyoseiteki sokumen karano hyoka ni kansuru kenkyu*) in January 2000. This group examined the political utility of epidemiologic research from the perspective of social accountability (Table 2). At the same time, the JEA set up the ad hoc committee on ethical issues (chairperson: Yutaka Inaba). This committee prompted the JEA to release a public statement on the need to explain the relationship between epidemiologic research and personal information protection to the public (in Japanese, *Kojin joho hogo ni kanrensuru ho seibi ni kansuru seimei*) in March 2000.^14^^,^^15^ This statement led to a degree of controversy when a group of citizens, including several lawyers representing patient rights, requested that the JEA provide a clear description of their rationale.

**Table 2.** Epidemiology in the 21st Century: A proposal of requirements.

1. To comply with relevant laws and guidelines including personal information protection
2. To conduct research in close collaboration with society
3. To utilize information adequately
4. To provide results that meet the expectation of society and further foster their trust
5. To conduct research responsibly as an epidemiologist
6. To present findings to society in a public way

These requisites were proposed by the MHW Working group on “Assessment of Epidemiologic Research in terms of Contributions to Health Policy” (April 2000) (in Japanese)

Soon after in April 2000, the Tamakoshi Group proposed a draft of guidelines for Informed Consent in Epidemiologic Research Version 1.0 (in Japanese, *Ekigaku kenkyu ni okeru infomudo konsento ni kansuru gaidorain*).^16^ This draft of guidelines, which focused on observational studies, stressed the apparent need for informed consent.

Following these events, in the fiscal year 2000, the Ministry of Health, Labour and Welfare (MHLW, formerly MHW) organized two nationwide efforts to examine the ethical implications of epidemiologic research: (1) An ad hoc committee under the auspices of the Advanced Medical Technology Assessment Panel of the Health Sciences Council examined privacy protection in scientific studies using epidemiologic methodology (hereinafter referred to as “the former ad hoc committee”; and (2) the MHLW Grant Research Project for the Study of Ethical Ramifications and Personal Data Protection in Researches and Programs Using Epidemiologic Methods which was led by Eiji Maruyama, a professor of law at the Kobe University Graduate School of Law (hereafter referred to as “Maruyama Group”). The Maruyama Group consisted primarily of clinicians and epidemiologists with a few experts in the fields of bioethics and law. Two members of the Tamakoshi group, Akiko Tamakoshi and Zentaro Yamagata, both epidemiologists, also joined the Maruyama Group.

In October 2000, the national committee in charge of the Personal Information Protection Bill announced the basic principles of the future bill (In Japanese, *Kojin joho hogo kihon hosei ni kansuru taiko*). Epidemiologists and the MHW, however, noted that the contents of this report, which were highly influenced by the Eight OECD Principles, could restrict epidemiologic research.^17^^,^^18^

## PART III: The Inter-Ministry Joint Committee proposes a set of guidelines (2001-2002)

As a result of the then-rising controversy surrounding the Personal Information Protection Bill (October 2000), the Seventh Division of the Japan Science Council announced a bulletin in March 2001 on “The Modality of Legislation Concerning the Protection of Personal Information in Medical Research ( in Japanese, *Igaku kenkyu kara mita kojin joho no hogo ni kansuru hosei no arikata ni tsuite*).”^19^ This bulletin proposed that certain conditions, including the duties prescribed for the “(commercial) handling of personal information” and the “fundamental principles” found in the Personal Information Protection Bill be excluded from medical research. Other organizations voiced similar opinions.^20^ Also in March 2001, the Association of Japanese Medical Colleges (AJMC) voted to create their own set of ethical guidelines in response to the continuing discussion of ethical guidelines for epidemiologic research.^21^

In April 2001, as the Diet failed to enact the bill for personal information protection, which was claimed to be a “restricting media law” by people in the mass media, the Maruyama Group proposed their draft, “Guidelines for Personal Information Protection and Bioethical Issues in Epidemiologic Research (in Japanese, *Ekigaku teki shuho o mochiita kenkyu nado niokeru seimeirinri mondai oyobi kojin joho hogo no arikata ni kansuru shishin*).”^22^ This draft was neither finalized nor put into effect partly because clinical researchers were concerned about its influence. However, the Maruyama group’s draft prompted the MHLW to create a second ad hoc committee for the development of ethical guidelines specific to epidemiologic research under the auspices of the Science and Technology Panel of the MHLW Health Sciences Council. Likewise, in August 2001, the MEXT created a sub-committee on epidemiologic research under the Council for Science and Technology. This same MEXT sub-committee was in charge of finalizing the draft created by the AJMC sub-committee (the AJMC also falls under the jurisdiction of the MEXT).

The MHLW ad hoc committee and the MEXT sub-committee combined to form the Inter-Ministry Joint Committee (IMJC) in September 2001. The IMJC consisted of clinical researchers, epidemiologists, lawyers, and media representatives. Through the collaborative efforts of these two ministries, which collected public comments and held a total of three meetings, the IMJC succeeded in publishing the Ethical Guidelines for Epidemiologic Research.^21^ This set of guidelines took effect in June 2002. Although these guidelines are not legally binding, both the MHLW and MEXT, which provide the bulk of government funding for scientific research, strongly recommend that all studies using epidemiologic methodology conform to these guidelines.

The JEA and its members have contributed to the development of the IMJC’s set of guidelines in several ways. Before the announcement of the ethical guidelines, the JEA announced ethical proclamation for epidemiologic research in January 2002 (Table 3). However, in an attempt to supplement this set of guidelines, the JEA developed their own set of guidelines in October 2002 (in Japanese, *Ekigaku kenkyu o jisshi suruniatatteno rinri shishin*). According to these guidelines, the JEA established an “Ethics and Review Board” in both eastern and western Japan.

**Table 3.** Ethical proclamation for conducting epidemiologic research by the Japan Epidemiological Association.

1. Research with the aim to reveal the truth
2. Research respecting the human rights of all subjects
3. Research using the most suitable methods to achieve its objectives
4. Research maintaining all social norms
5. Research always opening to society

(January 2002 http://wwwsoc.nii.ac.jp/jea/main/sengen.html accessed October 23, 2004, in Japanese)

## CONCLUSION: The need to assess the process of developing ethical guidelines

This report has reviewed a selection of historical events related to the development of the Ethical Guidelines on Epidemiologic Research published in 2002, which were the first ethics guidelines in this field. Although these guidelines had been planned to be revised after 5 years, the revision was facilitated and published in December 2004 because the Protection of Personal Information Act will take effect in April 2005. The authors did not address the revision process of these guidelines, but it needs to be examined elsewhere. Further studies are required to clarify problems specific to the contents of these guidelines. For instance, an in-depth study is needed that can critically evaluate the developmental procedures of guidelines. In particular, the problem of consistency among guidelines needs to be resolved. This is necessary not only for the proper administration of these guidelines, but to provide society with an agreed-upon set of guidelines.

Since the release of the Ethical Guidelines for Epidemiologic Research, the issue of personal information protection has increasingly gained attention. The Protection of Personal Information Act was enacted in May 2003 by a draft amendment of the Personal Information Protection Bill. This law states that scientific researchers are relieved of their duties prescribed for “the (commercial) handling of personal information,” but are, at the same time, obligated to release information to the public. This legislation, in other words, asks researchers to grapple with the development of ethical guidelines. Epidemiologists need to bear in mind that the development process of ethical guidelines should be an interdisciplinary and public effort. Yonemoto^23^ and Nudeshima^24^ have suggested a lack of social consensus regarding the development of medical research guidelines in Japan. This argument is based on the following perspectives: (1) original members of the governmental review board were primarily academics and experts selected by the national government; (2) the review process was not fully open to all interested parties; and (3) the board’s decision often does not reflect the views of all relevant parties. Time is insufficient to discuss all aspects of the issue, and each party has its own vested interests. Despite these limitations, however, an interdisciplinary approach can foster a more substantive and effective process for the development of guidelines. This review serves as a first step to raising the level of efficiency and efficacy of the development of future guidelines and their revision.

Ethical guidelines for epidemiologic research must ensure the highest standards of ethical research while also preserving the promotion and growth of our field. In developing effective guidelines, epidemiologists must address questions, including “What is epidemiology in terms of methodology?” and “What is its role in society?” Epidemiologists also need to convey their acquired knowledge to academics, professionals, legislators, the media, and the general public.^25^ Only by facilitating an informative relationship with society, will epidemiologists be able to cultivate greater understanding and acceptance of this field.
